# Supplementary figures and images for: Schistosoma japonicum cathepsin B2 (SjCB2) facilitates parasite invasion through the skin
Source: PLoS Negl Trop Dis. 2020 Oct 26;14(10):e0008810. doi: 10.1371/journal.pntd.0008810 (PMC7644097; doi:10.1371/journal.pntd.0008810)

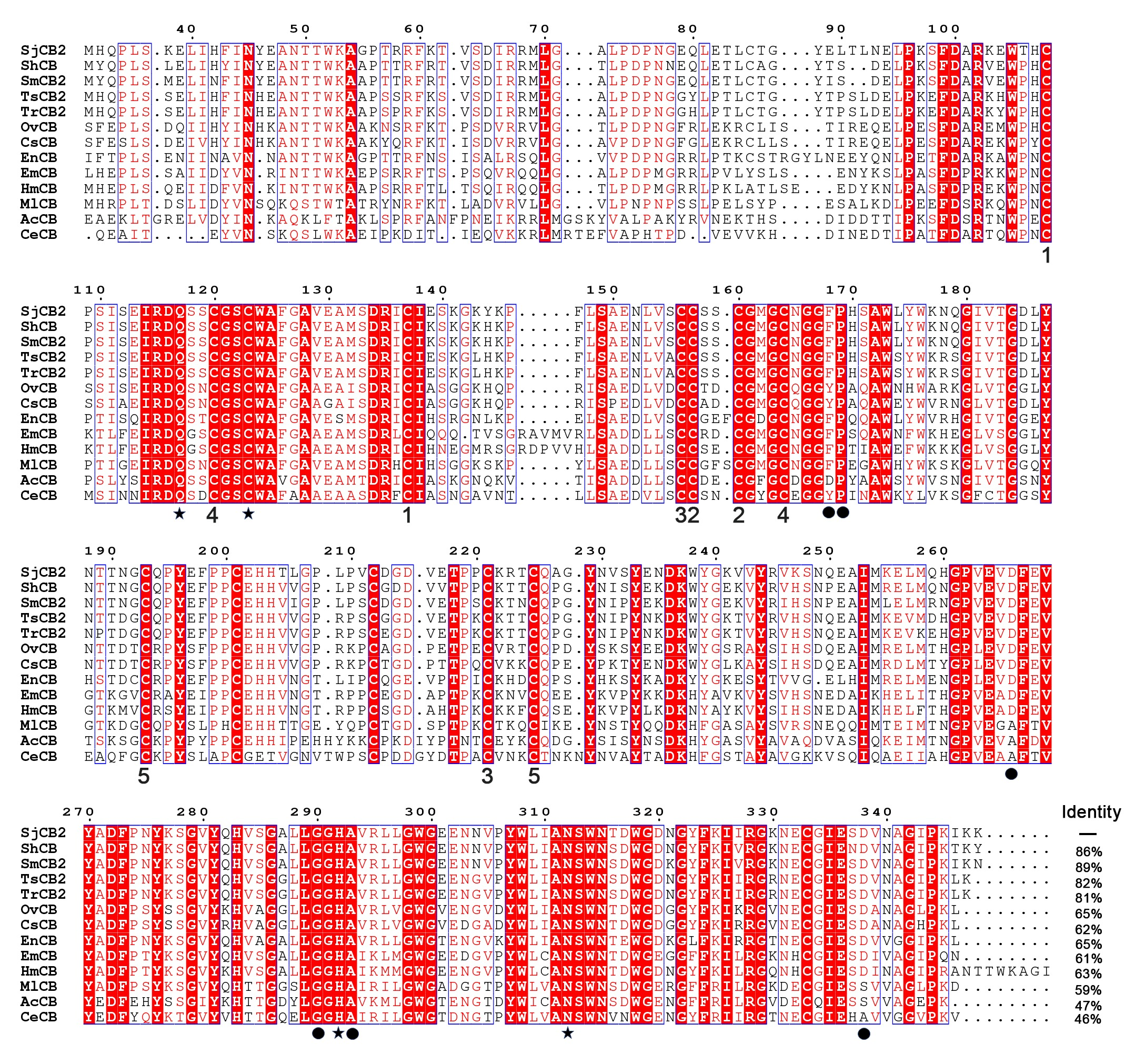

Supplement: S1 Fig — Predicted N-glycosylation sites are highlighted in grey. The active site residues are indicated by the five-pointed star, Cys123 and His292, forming a catalytic dyad; Gln312, preceding the catalytic Cys123, involved with the formation of the oxyanion hole; and an Asn117 residue which orients the imidazolium ring of the catalytic His292. The S2 subsite residues are indicated by the solid circle, they represent the dominant substrate specificity subsite of papain-like cysteine proteases. Cys residues of the pept_C1 domain that are predicted to form the disulfide bond are indicated by the same number. (TIF) [file pntd.0008810.s001.tif]

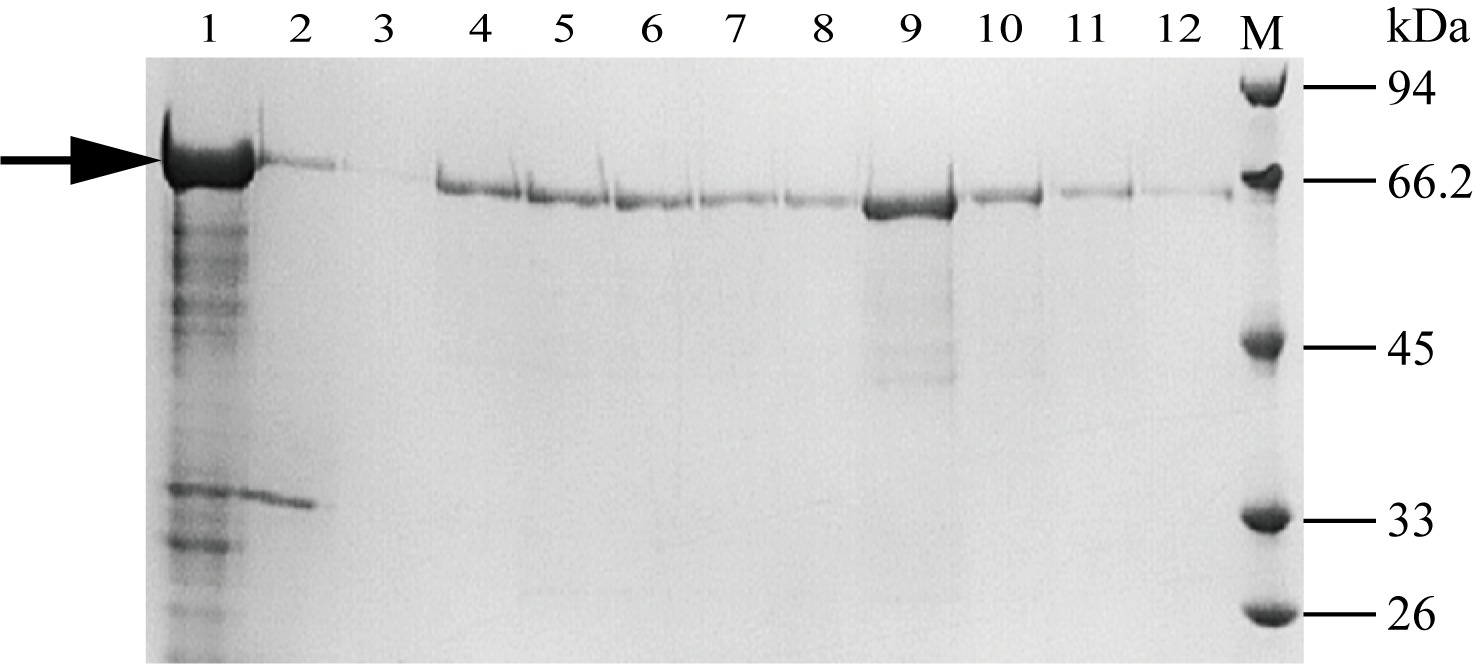

Supplement: S2 Fig — Lane 1: soluble fraction from E. coli cell lysates; Lane 2–3: column flow through; Lanes 4–8, 50 mM imidazole eluent; Lanes 9–12, 250 mM imidazole eluent; M, PageRuler Prestained Protein Ladder (Thermo Fisher Scientific). Arrowhead indicates rSjCB2. (TIF) [file pntd.0008810.s002.tif]

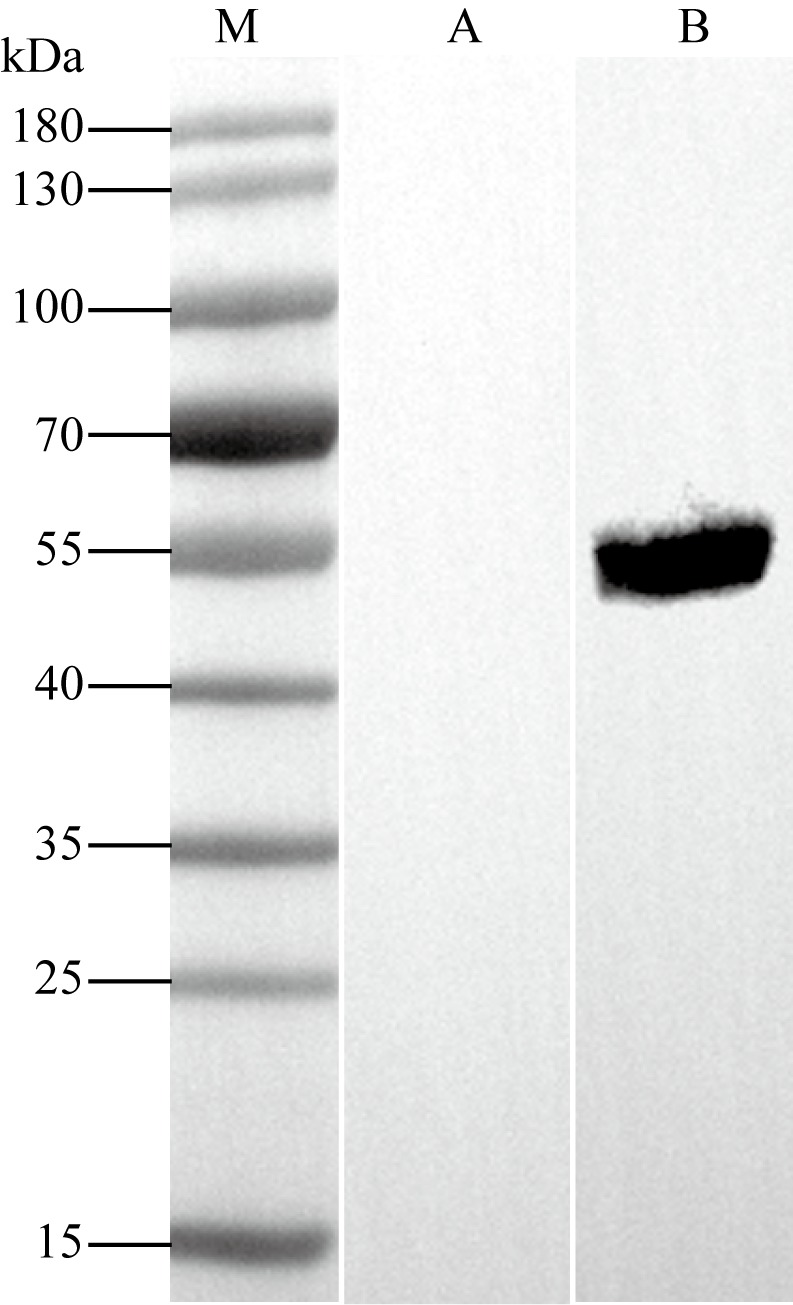

Supplement: S3 Fig — ES proteins of 42 dpi adult worms were resolved by SDS-PAGE, blotted onto PVDF membrane, and detected by pre-immune rabbit IgG (A) or anti-rSjCB2 IgG (B). (TIF) [file pntd.0008810.s003.tif]
