# Supplementary material for: Schistosoma japonicum cathepsin B2 (SjCB2) facilitates parasite invasion through the skin
Source: PLoS Negl Trop Dis. 2020 Oct 26;14(10):e0008810. doi: 10.1371/journal.pntd.0008810 (PMC7644097; doi:10.1371/journal.pntd.0008810)
Supplement: Attachment — Submitted filename: Response to Reviewers.docx [file pntd.0008810.s006.docx]

Editor Comments to Author:

Thank you very much for submitting your manuscript "*Schistosoma japonicum* cathepsin B2 (SjCB2) facilitates parasite invasion through the skin" for consideration at PLOS Neglected Tropical Diseases. As with all papers reviewed by the journal, your manuscript was reviewed by members of the editorial board and by several independent reviewers. The reviewers appreciated the attention to an important topic. Based on the reviews, we are likely to accept this manuscript for publication, providing that you modify the manuscript according to the review recommendations.

*Thank you for giving us the opportunity to improve this manuscript. We have addressed all the comments from all the reviewers, please see our point-by-point response below.*

Katja Fischer

Guest Editor

PLOS Neglected Tropical Diseases

Michael Hsieh

Deputy Editor

PLOS Neglected Tropical Diseases

Reviewer Comments to Author:
Methods

Reviewer #1:

In the section of Antiserum of rSjCB2 inhibited the invasion of *S. japonicum* cercariae. It seems to me that the rSjCB2 antiserum includes antibodies against GST and SjCB2, thus it does not provide a convincing result for the function of SjCB2, as the GST protein is also *S. japonicum* originated.

*Response: Thank you. We are sorry for using the wrong words. In fact, all the experiments involving antibody using used the purified anti-rSjCB2 IgG (including immune-blotting, immunofluorescence and antibody inhibition of the cercariae invasion). The IgG of GST has been cleared by incubating with GST protein (lines 202-205), as showed by the ELISA test against GST protein (S2 table).*

Also, the design of the experiment is questionable as water but not buffer was used for the binding reaction between antibodies and the parasite-released SjCB2. Antibody-antigen binding usually is most efficient in aqueous buffers at physiological pH and ionic strength, such as in phosphate-buffered saline. Without buffer, the affinity between antibodies and antigen is low. The authors should redesign and re-perform the experiment.

*Response: Thanks for your comments. However, we did use PBS as the buffer of pre-immune IgG and the anti-rSjCB2 IgG. We have added this information in the revised manuscript (lines 370-371). We worried that PBS may affect the vitality of S. japonicum cercariae, so we added the water control. The results showed that PBS didn’t affect the vitality of S. japonicum cercariae at all, because there was no difference in worm number between the water control and the pre-immune IgG control. We have modified figure 8 to show the results more accurately.*

Reviewer #2:

Methods are appropriate to study aims. No real concerns, however clarification is required.

*Response: Thank you, clarifications were provided below.*

Expression and purification of ySjCB2- Presumably deglycosylation is not part of purification process? If so, please write the method for deglycosylation separately from purification.

*Response: Thank you so much for your careful check. We have rewritten this part separately from the purification process (lines 291-297).*

Degradation of host proteins- It is noted that 500 nM of purified ySjCB2 was used is digestion of tested substrates. This is approximately 5 fold higher than the maximum enzyme concentration used in the enzyme activity assay (80-100 nM, Fig 5A). Did the authors test out a range of enzyme concentrations for digestion of all substrates tested?

*Response: Thank you for your comments. Actually, the maximum enzyme concentration against the fluorogenic peptide substrate Z-FR-MCA was around 160nM (Fig 5A). Because enzyme has different affinities for different substrates, so for the macromolecular natural substrates’ digestion experiments, we used a higher enzyme concentration to ensure that all the selected substrates can be degraded. We didn’t test the range of enzyme concentrations for digestion, but we tested the range of the incubation time and found that after 18 hours of incubation, all the substrates were degraded.*

Why did the authors not include inactivated ySjCB2 as a negative control, eg. By using E-64 or heat inactivation as in the cultivated human digest assay.

*Response: We appreciate for your valuable comment. On the one hand, we want to save the usage of ySjCB2, because it’s hard to obtain; on the other hand, we referred to the methods in references 42 and 43, which didn’t use inactivated enzyme as negative control, but added the reaction buffer equal volume of the enzyme. We believed this method could achieve the same purpose.*

Inhibition of the cercariae invasion- How did the authors decide to use 10 min incubation period with SjCB2 antiserum? Did the authors test a range of incubation times?

*Response: Thank you for your comment. This is a suitable incubation time determined by our observation. In the S. japonicum cercariae infection experiment, we added 20 μL water or antibody solution on a small cover slip, and added 40 cercariae. We found that after 10 min incubation with water or antibody, the vitality of the cercariae wouldn’t be affected. But when it exceeded 10 min, the evaporation of water gradually increased, and the vitality of the cercariae gradually decreased. So we thought 10 min was a suitable incubation time for antibody binding, at the same time, the vitality of the cercariae wouldn’t be affected.*

Was the serum incubated with cercariae at RT or at 37℃?

*Response: The incubation was performed at room temperature.*

Reviewer 3:

(No Response)

Results

Reviewer #1:

(No Response)

Reviewer #2:

General comment – Authors should use full name or include explanation of acronyms of enzymes when referring to them for easier reading. For example, Line 389- SjCB2 is closely related to SmCB2 and ShCB sharing the same branch (Fig 1B). The explanation for what SmCB2 and ShCB are only described in the figure legend. e.g. in line 407. The use of genus and species should be consistent, with the name written in full when used for the first time. Please amend throughout the manuscript as appropriate.

*Response: Thank you for pointing this out. All corrected.*

Fig 3 – Please include arrows indicating the cercariae heads panel D, H, L. It would be visually helpful if there is a diagram of acetabular glands and their ducts of *S. japonicum* cercariae or indication of these structures in the scans.

*Response: Thank you for your good advice. Arrows indicating acetabular gland and the gland duct were added in the updated Figure 3.*

Is purified recombinant ySjCB2 used in this study in glycosylated form as produced in *Pichia pastoris*? Will this affect its enzymatic activity? Was the sequence of purified ySjCB2 confirmed by N-terminal sequencing?

*Response: Thank you for your comments. Sorry for not being clear enough. The enzymatic analysis was used the deglycosylated ySjCB2 in native condition. We have revised the deglycosylation part in the Methods (lines 291-297). The expressed ySjCB2 was verified by immune-blotting against anti-rSjCB2 IgG.*

Degradation of host proteins- Fig 6. To standardize the results, all samples should be run on the same gel and includes protein ladder to mark sample size.

*Response: Thanks for your suggestions. Because the substrates were bought at different times, so the degradation experiments weren’t performed at the same time. In order to present the results more accurately, we have added a protein ladder to the electropherogram of each substrate, please see our updated Figure 6.*

Fbn, IgA and C3 gels are very faint to visualize.

*Response: Thank you. Fbn, IgA and C3 were bought in solution, protein concentration was much lower than others. We have used as higher concentration of proteins as possible in the degradation experiments, compared to the controls, these three proteins were obviously degraded by ySjCB2.*

Reviewer #3:

(No Response)

Conclusions

Reviewer #1:

Line 135-6, whether it is appropriate to state ‘and also offered a new target for novel anti-schistosomal therapeutics’ is questionable, as no functional studies on SjCB2 in the intra-mammalian developmental stage have been carried out in the current work.

*Response: Thanks for this suggestion. The sentence has been corrected to ‘and also offered a new target for novel anti-infection therapeutics.’ (lines 136-137).*

Reviewer #2:

Line 553-555 – Please confirm the author’s statement accurately reflects what is reported in the referenced article 32.

*Response: Thank you for your careful checking, and sorry for using the wrong reference, which has been deleted already.*

ySjCB2 appears to be promiscuous with a wide range of substrates, ranging from collagen, elastin, fibrinogen, IgM, C3, hemoglobin, etc. Are there any other cercariae’s cathepsin Bs that digest a wide range of proteins?

*Response: Yes, there are two other cercariae’s cathepsin Bs can hydrolyze multiple substrates. Schistosoma mansoni cathepsin B2 (SmCB2) also can digest a range of substrates, including collagen, fibronectin, keratin, immunoglobin G and complement C3 (see reference 32); Trichobilharzia regenti cathepsin B2 (TrCB2) can degrade collagen, elastin, fibrinogen and haemoglobin (see reference 28).*

Could the authors include possible reasons for many of the various skin components individually tested with ySjCB2 did not appear in the results from cultivated human epidermis?
Only Keratin was apparent in the analysis in Table 1, but not collagen, fibronectin etc.

*Response: Thank you. This is because the skin material was limited. We were unable to get human skin from the hospital, so we have to use the cultivated human epidermis, which was differentiated from the keratinocytes. Collagen, elastin and fibronectin are main structural proteins in the dermis, so we couldn’t see these proteins appeared in the proteomic data of skin digestion.*

Could the authors speculate that if cercariae were treated with general cysteine protease inhibitor such as E-64, will reduction in cercariae invasion in the mouse experiment be more profound that using anti-ySjCB2 serum?

*Response: Thanks for your suggestion. Yes, you are right. Actually, we did this experiment, after incubation with E-64, the cercariae invasion ability had a 47% reduction, which was more profound than the antibody inhibition. Since E-64 was a chemical compound and a general cysteine protease inhibitor, it can inhibit many isoforms of cathepsins, not only SjCB2. Besides, cercariae was sensitive to compounds. So the higher inhibition rate of E-64 may be because the vitality of cercariae was affected by this compound, or other cysteine proteases were also involved in the S. japonicum cercariae invasion. However, our goal was to determine the contribution of SjCB2 to the invasion of S. japonicum cercariae. So we decided to use the anti-rSjCB2 IgG to bind the native protein released by cercariae, it’s more specific than E-64. At the same time, we found the antibody solution (anti-rSjCB2 IgG and pre-immune IgG) had no effect on the cercariae’s vitality (updated Figure 8). Therefore, we think anti-rSjCB2 IgG can be more suitable to accurately evaluate SjCB2’s contribution to the S. japonicum cercariae invasion process.*

Line 670- 677. Considering that there would be a family of proteases required to work together for host invasion, evident by only 22% reduction in cercariae infection with anti-ySjCB2 treatment in mice, would targeting specific protease such as ySjCB2 be useful in developing anti-invasion therapeutics?

*Response: Thanks for this constructive suggestion. Actually, we believe this protease not only played a role in the skin penetration, but played more important roles in resisting the host’s immune attacks to help the invading larvae surviving in the mammalian hosts. We are planning developing anti-infection therapeutics targeting this enzyme.*

Reviewer #3:

(No Response)

Editorial and Data Presentation Modifications

Reviewer #1:

Minor Revision

*Response: Figures 3, 6 and 8 were modified to present the results more accurately.*

Reviewer #3:

(No Response)

Reviewer #3:

(No Response)

Summary and General Comments

Reviewer #1:

In this study, Zhu et al. reported bioinformatics characterization, localization, biochemical features and functions of a cathepsin B cysteine protease derived from *Schistosoma japonicum*, SjCB2, and explored the function of the enzyme by cercariae invasion experiment in a murine model. The current study is a piece of work that follow up the previous studies on SmCB2, however, it does provide some feature of its own based on robust data. The results and discussion were presented in an appropriate manner.

*Response: We appreciate your positive evaluation to our manuscript.*

Major concerns

It is quite confusing that the authors produce the anti-rSjCB2 antibody using a GST-fused protein, which requires additional step to get rid of anti-GST antibody later on prior to application? So why not express the protein with a His-tag only in *E. coli*, or use the recombinant SjCB2 protein produced in *P. pastoris*.

*Response: Thanks for your comments. Actually, we spent a long time and tried different methods to express this protein. At the beginning, we used E. coli system and tried different vectors. Firstly, we used the PET28a vector, which has a His tag, but we didn’t detect the expression of the protein. Then we decided to use the pGEX-h-6t-1 vector, which has both GST tag and His tag, the protein was successfully expressed, but in an insoluble form. So we purified rSjCB2 with Ni^2+^-NTA affinity-chromatography. In order to save time, we decided to use the purified rSjCB2 to prepare antibody. But rSjCB2 contained a GST tag, so we purified GST protein to remove the anti-GST IgG. Finally, we tried the eukaryotic expression system – P. Pastoris, and successfully expressed the active ySjCB2, and used for the enzyme activity analysis.*

Line 200, the author stated that ‘incubating the serum with purified GST protein’, I wonder whether the affinity-purified IgG was further incubated with GST protein, otherwise, in the line 201, it should be described as ‘without anti-GST antibody (not only IgG, but also other isotypes). In the current format, the recombinant SjCB2 is actually GST-rSjCB2, the IgG titers tested in the two immunized rabbits are against GST-rSjCB2.

*Response: Thank you for this comment. We are sorry for not describing this process accurately. However, after obtaining the rabbit serum, we firstly used the Protein G-Sepharose to purify the IgG part, then the affinity-purified IgG was further incubated with purified GST protein to remove the anti-GST IgG. So the SjCB2 antibody used in all experiments was only the IgG isoform (of anti-rSjCB2 IgG). We have revised this part as ‘The serum was collected at day 53. IgG was isolated from the collected serum using Protein G-Sepharose (1ml) as described [25] and stored at -20℃. Because there is a GST tag in the recombinant SjCB2, we removed the anti-GST IgG by incubating antibody with purified GST protein at 4**℃.’ (lines 200-205).*

In the methods section, In the immunoblotting, the authors stated that ‘anti-rSjCB2 polyclonal IgG diluted 1:1000’, while in the immunofluorescence assay, SjCB2 antiserum in blocking solution with a concentration of 1 μg/ml. ‘anti-rSjCB2 polyclonal IgG’ or ‘SjCB2 antiserum’ are quite ambiguous without appropriately described in the methods section. In the results, the authors stated that ‘Immunoblotting was used to determine whether rabbit antiserum’, and ‘Immunofluorescence microscopy on fixed cercariae using affinity purified polyclonal antibodies raised against rSjCB2’, which are conflict with those described in the methods. Noting that the purified polyclonal antibodies and antiserum are different concepts. Same problem: Line 449, using affinity purified polyclonal antibodies while in Line 456, rabbit polyclonal primary antibody was used. What are you used exactly? For the later one, purified or not purified? Line 441, ‘sample incubated with the pre-immune rabbit serum’ while Line 444 ‘visualized by normal rabbit IgG’. Check these phrases throughout the manuscript. Rigorous and clear expression is definitively needed.

*Response: Thank you so much for your careful check, we are so sorry for using the ambiguous words that confused the readers. However, purified anti-rSjCB2 IgG was the only antibody used in all related experiments, including immune-blotting, immunofluorescence and antibody inhibition of the cercariae invasion. All the ambiguous words were corrected in the revised manuscript.*

In the section of Antiserum of rSjCB2 inhibited the invasion of *S. japonicum* cercariae.
It seems to me that the rSjCB2 antiserum includes antibodies against GST and SjCB2, thus it does not provide a convincing result for the function of SjCB2, as the GST protein is also S. japonicum originated. Also, the design of the experiment is questionable as water but not buffer was used for the binding reaction between antibodies and the parasite-released SjCB2. Antibody-antigen binding usually is most efficient in aqueous buffers at physiological pH and ionic strength, such as in phosphate-buffered saline. Without buffer, the affinity between antibodies and antigen is low. The authors should redesign and re-perform the experiment.

*Response: ‘the rSjCB2 antiserum’ here actually was ‘the anti-rSjCB2 IgG’, and we did use PBS as the puffer of pre-immune IgG and anti-rSjCB2 IgG. For more details, please see our answer to your same question in the Methods part.*

Which form of ySjCB2 was used for the determination of enzyme activity and degradation of host proteins? The deglycosylated one or the one without deglycosylation?

*Response: We used the deglycosylation one. We have added more detailed description of the deglycosylation process in the Methods (lines 291-297). Deglycosylation of ySjCB2 was performed under denaturing and native conditions, the former was used in the SDS-PAGE analysis, the latter was used in all experiments that needed the active enzyme.*

I suggest that authors should determine the expression profile of the gene throughout different developmental stages, which can further provide the insights on the molecule. If not at least discuss the work by Liu et al (PLoS Comput Biol. 2014 Oct; 10(10): e1003856.), in which it shows that the relatively highest expression of SjCB2 is in the adult male worms, indicating a different functional cue for the gene.

*Response: This is an excellent point. We are sorry we don’t have the materials to supplement this experiment. However, we read the work by Liu et al (PLoS Comput Biol. 2014 Oct; 10(10): e1003856.) and checked the mRNA expression in 14 to 28 dpi old worms from our previous published transcriptomic data (Nat. Commun. 2017;8:14693.), we found that SjCB2 was stably and highly expressed during these stages. Besides, we detected SjCB2 in the ES products of adult worms by immune-blotting (S3 Fig). So we speculated that SjCB2 was a multifunctional protease, which not only facilitates cercariae penetration, but also may involves in degrading blood or blood clotting components in the adult worms. We have added these information into the discussion (lines 631-632, and 672-673).*

Line 96, Adult schistosome worms reside. Line 97-98, noting that different *Schistosoma* species have different egg yields per day per pair. Also, Line 98, ‘particularly the liver,’ refers to the infection of *S. mansoni* and *S. japonicum*, but not *S. haematobium*.

*Response: ‘Adult schistosome reside’ corrected to ‘Adult schistosome worms reside’ (line 96); ‘Adult schistosome reside in the veins as male/female pairs producing hundreds of eggs per day’ corrected to ‘Adult schistosome worms reside in the veins as male/female pairs producing hundreds to thousands of eggs per day’ (lines 96-97); ‘Eggs can become entrapped in various host tissues, particularly the liver, and induce granulomatous reactions that are regarded as the main pathogenic factor of schistosomiasis’ corrected to ‘Eggs can become entrapped in intestines or liver (for S. mansoni and S. japonicum) or bladder and urogenital system (for S. haematobium), and induce granulomatous reactions that are regarded as the main pathogenic factor of schistosomiasis’(lines 97-100).*

Lines 115, serine protease, and cercarial elastase. Noting that the references 17-21 also include the work from *S. japonicum*, while the sentence refers the enzymes from *S. mansoni.*

*Response: Thank you for your careful check. The references of cercarial elastases from S. japonicum were deleted (references 20 and 21).*

**Minor ones:**

Line 123, may be both

*Response: Corrected (line 124).*

Line 128, a full name required for *P. pastoris*

*Response: ‘P. Pastoris’ replaced with ‘Pichia Pastoris’ (line 129).*

Line 130, blood circulatory system?

*Response: ‘blood system’ changed into ‘blood circulatory system’ (line 131).*

Line 132, Immunochemistry or Immunofluorescence?

*Response: Immunofluorescence, corrected (line 133).*

Line 286, what does it mean that ‘*S. japonicum* cercariae were isolated from Anhui province, China’.

*Response: I think you referred to line 147, not 286. Thanks for your comment, we revised the sentence as ‘A Chinese mainland isolate of S. japonicum from Anhui province, and the infected Oncomelania hupensis snails were provided by the pathogen biology laboratory of the National Institute of Parasitic Diseases, Chinese Center for Diseases Control and Prevention, Shanghai.’* *(lines 148-152).*

Line 311, anti-GST antibody

*Response: I think you referred to line 199, not 311. ‘GST antibody’ changed into ‘anti-GST IgG’ (line 203).*

Line 369, the open reading frame (ORF) of SjCB2 is 1,048 bp in length and encodes a preproenzyme of 348 amino acids. 348 multiply 3 is 1044bp, if add the stop codon is 1047bp.

*Response: Thanks for your comment. The sentence has been corrected to ‘The open reading frame (ORF) of SjCB2 is 1,047 bp in length and encodes a preproenzyme of 348 amino acids with predicted molecular weight of 39.5 kDa and isoelectric point of 6.48.’ (lines 381-383).*

Line 378, degrade blood components??

*Response: ‘degrade blood’ corrected to ‘degrade blood components’ (line 390).*

Line 444, detected by normal?

*Response: ‘visualized by normal rabbit IgG’ corrected to ‘detected by pre-immune rabbit IgG’ (lines 457-458).*

Line 477, data were

*Response: Corrected (line 490).*

Line 498, natural macromolecular substrates?

*Response: ‘macromolecular natural substrates’ corrected to ‘natural macromolecular substrates’ (line 512).*

Line 642, facilitate cercariae penetration?

*Response: ‘facilitate cercariae migration’ corrected as ‘facilitate cercariae penetration’ (line 660).*

Line 682, blood components

*Response: ‘digesting blood’ corrected to ‘digesting blood components’ (lines 700-701)*

Reference should be follow the style of the journal, and all genus names in references should be italic.

*Response: All Corrected.*

Reviewer #2:

This study investigate the role of cathepsin B2 (SjCB2) of *Schistosoma japonicum* for host invasion. Recombinant SjCB2 was expressed, purified and utilized to address its function. SjCB2 appeared to digest a wide range of host proteins including skin, blood and innate immune components. However, further studies should be conducted to confirm these findings and to investigate SjCB2 specific function.

*Response: We appreciate your positive evaluation to our manuscript. We are designing experiments to confirm these findings and to investigate SjCB2 specific function.*

Reviewer #3:

The study has been designed well and have addressed key aspects of the potential activities of the proposed parasitic protease with the help of previous publications.

*Response: We appreciate your positive evaluation to our manuscript.*

**Major comments:**

Line 187 - Imidazole concentrations of the Ni elution buffers were not mentioned and no information of whether the eluted proteins are dialyzed into a new buffer. Depending on that, if the imidazole concentration exceeds the Bradford assay comparability, the use of Bradford assay may not be accurate for the protein quantification. Dialysis into a new buffer could also result in protein precipitation. So it’s better to address this in the manuscript in a sensible manner.

*Response: Thanks for your suggestion, we used an Amicon Ultra-10 device to concentrate the protein. At the same time, we used this device to exchange the protein buffer (buffer exchange and protein concentration can be both accomplished in this device). After protein concentration and buffer exchange, the concentration of the imidazole was about 10 mM, and this wouldn’t affect the Bradford assay. We have rewritten this part in the manuscript (lines 189-193).*

Line 175 - Methods doesn't mention about the inclusion of the GST tag in the cloning. But later (Line) mentioned about the presence of a GST tag. It should be mentioned in the cloning section.

*Response: Thank you for pointing this out, we have added this part into the manuscript (lines 185-187).*

Line 503 - Author has mentioned "All the selected protein substrates were efficiently degraded by ySjCB2". This statement needs more clarification. It’s has only been done with visual detection and has not used any band intensity measuring software, therefore the level of efficiency is very wage. In fact, the band in the albumin and IgG are has considerably similar intensities between the - and +. In addition, mentioning of the resulted fragments in a denaturing gel for each of the protein used with their respective molecular weight would help to explain the resulted bands in each of + lanes. Therefore, this section of results needs to be thoroughly explained. Furthermore, addition of a protein ladder for the image is required.

*Response: Thanks for your comments. We are sorry for the improper word using and unclear result statements, we have rewritten this part as ‘Collagen, elastin, keratin, fibronectin, immunoglobin A (heavy chain), immunoglobin M, complement C3 and hemoglobin were efficiently degraded by ySjCB2. Immunoglobin G and albumin could also be cleaved, but the effect was not obvious (Fig 6).’ (lines 517-520). Besides, we have added a protein ladder to the electropherogram of each substrate (see our updated Figure 6).*

Line 569 - The explanation given for obtaining two bands for rSjCB2 for the whole-body extract would be a one possibility. The other possibility might be, even though there is single SjCB2 there could be more Sj cathepsin Bs. Reference 35 suggests there might be 3. Can authors explain how they can exclude the cross reactivity of the rSjCB2 antibody with closely related Sj other cathepsins. This comment also related to the localization results, but that could be excluded since the acetabular glands are the only localized site.

*Response: Thank you. Cathepsin B is first synthesized in a zymogen form, the pro-peptide domain needed to be excised in order to fully active the enzyme. Therefore, there are two forms of SjCB2 existed in the cercariae extracts (zymogen and activated enzyme). We also blasted the protein sequence of SjCB2 against the latest S. japonicum proteome database, and found that another cathepsin B (NCBI ACCESION: TNN04783.1), which has the highest homology to SjCB2, showed an identity of 52%. This identity was not high in a gene family. The molecular weight of this cathepsin B is 38.6 kDa, which is very close to SjCB2 (39.5 kDa). Therefore, the two bands in the immune-blotting analysis most likely represented the zymogen and the active enzyme form of SjCB2.*

**Minor comments:**
Line 195 - The site of infection is needed to be mentioned.

*Response: I think you mean the injection site, corrected as suggested (line 197).*

Line 198 - Correction from G-separate into G-sepharose

*Response: Corrected (line 201).*

Line 222 - homogenization method and conditions or a reference to a followed method is needed.

*Response: Homogenization method and conditions were added (lines 225-227).*

Line 264 - Method for the preparation of the P. pastoris X-33 strain in brief or a reference to a followed method is needed.

*Response: A reference (reference 25) was added (lines 269-270).*

Line 266 - Electroporation conditions are needed.

*Response: Electroporation conditions were added (lines 271-272).*

Line 267 - Please check the font size

*Response: Corrected.*

Line 291 - SI unit of for the microliter need to be corrected.

*Response: Corrected.*
